# Supplementary material for: Are carbon emissions trading and green financial instruments synergistic? -Comprehensive quantitative research based on content analysis
Source: PLoS One. 2024 Mar 7;19(3):e0298601. doi: 10.1371/journal.pone.0298601 (PMC10919601; doi:10.1371/journal.pone.0298601)
Supplement: S1 Data — (ZIP) [file pone.0298601.s001.zip › Data/地级市Z_F工作报告.zip!/┴╔─■/┤≤┴1⁄4╩╨/└┤╫╘╣1⁄2╓┌║┼í╛┬φ┐╦╩2╛▌═°í┐_2019.docx]

各位代表：

　　现在，我代表市政府向大会报告工作，请予审议，并请市政协各位委员提出意见。

　　一、2018年政府工作回顾

　　2018年是全面贯彻党的十九大精神开局之年，是改革开放40周年。在省委、省政府和市委的坚强领导下,我们坚持以习近平新时代中国特色社会主义思想为指导，全面贯彻党的十九大作出的战略部署，时刻牢记习近平总书记对辽宁、对大连的亲切关怀，坚持稳中求进工作总基调，认真践行新发展理念，按照高质量发展要求，大力开展“重实干、强执行、抓落实”专项行动，践行“严细实”工作作风，保持定力，积极作为，战胜了各种风险和挑战，加快推进“两先区”建设，统筹抓好稳增长、促改革、调结构、惠民生、防风险各项工作，较好完成市十六届人大一次会议确定的年度目标任务，实现新一届政府良好开局。

　　——我们攻坚克难促发展，经济运行健康平稳。预计全市地区生产总值增长6.5%左右；一般公共预算收入增长7%；固定资产投资增长10%左右；社会消费品零售总额增长8%左右；外贸进出口额分别占全省的65%和57%，超额完成省计划安排指标；城乡居民人均可支配收入分别增长7.8%左右和8%左右，居民消费价格指数涨幅控制在3%，城镇登记失业率为2.58%。经济运行“稳”的基础在巩固、“进”的势头在延续，高质量发展迈出坚实步伐。

　　——我们凝心聚力优环境，重大项目加快推进。狠抓营商环境建设，建立“服务秘书”制度，对全市重大项目进行全流程服务保障。全市推进实施重大项目177个，英特尔非易失性存储器项目二期工程正式投产，创造了英特尔工厂建设和投产全球新纪录，体现了“大连速度”和“大连效率”。恒力2000万吨/年炼化一体化项目投料开车，刷新了同行业同体量项目最快建设纪录，成为国家七大石化产业基地中推进速度最快的项目，标志着长兴岛世界级石化产业基地建设取得重大成果。

　　——我们坚定不移强实体，工业经济强劲增长。预计全市规模以上工业增加值、规模以上工业利润和工业投资分别增长15%、23%、40%，在全国副省级城市中名列前茅。我市被国务院评为“推动中国制造2025、促进工业稳增长和转型升级成效明显市”，金普新区上榜“2018中国工业百强区”。实体经济持续壮大，夯实了大连加快全面振兴的基石。

　　——我们标本兼治抓环保，生态环境显著改善。坚决打好污染防治攻坚战，空气质量达标天数317天，占全年的86.8%，比上一年增加17天；城市核心区6条8段黑臭水体得到彻底整治，在全省第一个通过生态环境部、住建部专项验收,我市入户问卷调查市民满意率达到96%。大连环境质量迈上新的高度。

　　——我们坚持不懈补短板,民生工程取得实效。推进62个蔬菜直通车进社区运营，建成6480个“菜篮子”零售网点。启动天然气入连工程，如期建成高压管道“东北线”，完成通气10万户。狠抓城市供热质量，实现“一下降两提高”。全力解决“办证难”“回迁难”问题，24个项目、22165户家庭产权证办理问题得到解决，2 个项目、2115户居民回迁安置工作全面启动。15项重点民生工程圆满完成，让城市更有温度、人民更加幸福。

　　——我们驰而不息转作风,行政效能持续提升。过去的一年，我们遇到的大事多、难事多。面对经济下行压力加大的复杂严峻形势，我们沉着应对、科学施策；面对干部人事冻结带来的人员不足、工作量较往年大幅增加等困难，我们苦干实干、团结拼搏；面对“温比亚”台风、连续罕见高温极端天气等严重自然灾害和非洲猪瘟疫情，我们同心协力、众志成城。全市上下始终以昂扬的精神风貌、强烈的责任担当、深厚的为民情怀，直面矛盾不回避，解决困难有办法，关键时刻冲得上、打得赢，确保了经济平稳健康、社会和谐稳定、人民安居乐业的良好局面。

　　一年来，我们主要做了以下工作：

　　（一）着力推动经济高质量发展

　　制造强市建设扎实推进。完善“1+X”政策体系，支持制造业创新中心等六大工程建设。石化行业加快向全产业链发展，恒力2000万吨/年炼化一体化、150万吨/年乙烯等项目加快了上下游产业布局的步伐，炼油业比重下降至46%左右，“油头重化尾轻”的产业结构得到改善。装备制造业加快向中高端迈进，重工•起重研制的全球首支世界最大2.2万标箱船用曲轴、大连造英菲尼迪汽车、大机车首列新一代中低速磁浮车成功下线，光洋智能制造产业园、红沿河核电二期工程有序推进，中国一重“核电快堆”技术填补我国空白，东北首个海上风电项目实现并网发电，中船重工海上风电装备产业园投入使用。大力实施“三品”战略，大杨集团智能化改造一期项目完成，普兰店获“中国橱柜名城”称号。

　　新动能加快成长。强力推动战略性新兴产业发展，松下新能源汽车电池、华录集团超大容量蓝光存储研发及产业化等项目投产，融科储能电池核心技术取得重大突破。华为大连软件开发云累计运行项目7000余个，中国移动5G联合创新中心实验室挂牌。全市高技术产业、战略性新兴产业增加值分别增长36.4%和41.6%。

　　现代服务业持续优化升级。提高航运中心能级，东北亚矿石分拨中心、商品车分拨中心以及国际邮轮母港建设取得积极进展，国内首船期货原油在大连港交割，国内首笔铁矿石期货保税业务在大连港落地，商品车年吞吐量增长16.2%、创历史新高。强化物流中心功能，国家多式联运示范工程重点项目基本完工，瓦房店跨境电商物流产业园、铁成物流园入选国家优秀物流园区。大连广告产业园区晋升国家级。扎实推进金融中心建设，引进金融及融资类机构109家，金融业实现增加值占GDP比重9.4%。大商所实现铁矿石期货国际化，继续保持全球最大农产品期货市场地位。全力推进旅游业发展，成功举办中日旅游大会、赏槐会、啤酒节和服博会，全市旅游综合收入1440亿元、增长12.5%。

　　(二)着力打好三大攻坚战

　　防范重大风险能力持续增强。坚决贯彻落实防范化解债务风险各项政策措施，全面落实主体责任，深入进行债务清查，严格实行限额管理，有效化解风险隐患，超额完成省政府下达的化债任务。多方施策稳定企业债务风险，大力开展互联网金融和各类交易场所治理整顿，牢牢守住不发生系统性风险底线。

　　脱贫攻坚取得扎实成效。坚决贯彻习近平总书记关于扶贫工作的重要论述，制定扶贫协作三年行动方案，构建五级立体化帮扶机制，开展资金、产业、劳务、人才等帮扶行动，向5个受援地区提供财政资金4.1亿元，推动大樱桃种植、韩伟养鸡等产业项目在六盘水市落地，扶贫攻坚取得重大进展。大力开展我市低收入村精准扶贫，人均可支配收入达到13623元、增长14.2%，全市128个低收入村中有26个摘帽。

　　污染防治强力推进。巩固扩大蓝天保卫战成果，空气质量达标天数提高4.6个百分点；全面实施河(库)长制，13处县级及以上饮用水源水质达标率100%。狠抓环保督察反馈意见整改落实，中央环保督察反馈的1685件群众信访举报案件已经销号1594件；国家海洋督察反馈的66个问题已完成整改62个，完成进度93.9%；中央生态环保督察“回头看”交办的33批1352件信访案件，已经办结1304件。妥善解决一批突出环境问题，人民群众的满意度明显提升。

　　（三）着力推进创新创业创投中心建设

　　自创区建设取得新进展。全面落实省、市自创区建设实施意见和三年行动计划，自创区带动全市创新发展的增长极作用进一步彰显。高新技术企业突破1200家、增长45%；技术合同交易额170亿元、增长37%；科技成果转化数量增长75%；万人有效发明专利拥有量达18.5件，约是全国的2.1倍；新注册各类企业4.2万户、增长9.3%。

　　重大创新平台加快建设。中科院大学能源学院揭牌，大连化物所洁净能源创新研究院获中科院批复，大连先进光源预研项目启动实施，大连人工智能研究院、干细胞与精准医学研究院落成。精细化工国家重点实验室、工业装备结构分析国家重点实验室、大连理工大学辽宁重大装备制造协同创新中心建设加快推进。全市拥有市级以上创新平台967家。

　　科技创新能力显著提升。推进大众创业、万众创新，建设6个海外创新中心和面向东北亚的国际双创园，高新区推动双创政策落地获得国务院表彰。国家技术转移东部中心大连分中心及3个专利运营中心落户，700项科技成果在我市转化。成功举办国际专交会。加强科技创新创业孵化载体建设，市级以上科技企业孵化器达43家，备案众创空间64家。我市企业获第七届中国创新创业大赛一等奖。

　　人才队伍建设务实推进。成功举办中国海创周，全年引进高层次人才13人、紧缺人才392人、海外优秀专家469人。通过“一事一议”方式支持高精尖缺人才1500余人。创建国家级人力资源服务产业园，重点培养创新人才2000余人；博士后设站单位增至129家，在站博士后800余人。

　　（四）着力深化重点领域改革

　　供给侧结构性改革深入实施。扎实有效去产能，依法依规制定推动落后产能退出方案，对钢铁、水泥、电解铝、平板玻璃等行业落后产能进行严格排查清理。推进房地产去库存，商品房待售面积下降16%，去化周期降至11个月。贯彻国家减税降费政策，全年为企业及工商户减免税633.2亿元、办理出口退税207.4亿元。

　　营商环境进一步优化。推出“放管服”改革23项举措，取消调整487项行政职权，精简市级管理权限125项，办理时限压缩69.4%。全面实施“双随机、一公开”监管改革，我市跨部门跨层级实现“一次检查，全面体检”经验受到国务院通报表扬。全面推进“一网、一门、一次”改革，进驻市行政服务大厅的审批事项中，100项实现“零跑腿”，555项实现“最多跑一次”，536项实现网上办，分别占15%、84%和81%。全面实施“证照分离”改革，企业开办时间压缩至3.5天。社会投资类项目审批不超过75个工作日，实现“一口受理、一事不两询、一事不两跑、全程网上办”。推行行政审批告知承诺制改革，长兴岛探索企业投资项目承诺制审批经验做法在全国推广。

　　行政管理体制改革有序推进。坚决落实国家、省市机构改革决策部署，调整优化市政府组成部门，改革后市政府工作机构由45个精简到37个，精简比例17.8%。推进事业单位改革，改革后保留事业单位166个，精简比例92.1%。整合优化园区管理机构，改革后保留园区管理机构15个，精简比例66.7%。

　　国资国企改革不断深化。燃气集团股权多元化改革顺利完成，棒棰岛宾馆集团、旅游集团、星海湾开发建设集团、检验检测集团正式组建，大化集团重整等改革加快推进。瓦轴、三寰、建投被确定为全国“国企改革双百行动”企业，22家驻连央企“三供一业”分离移交协议全部签订。

　　民营经济活力持续增强。召开民营企业家座谈会，成功举办“2018民营企业家大连行”活动。着力解决中小企业融资难问题，举办各类银企对接活动93场，市公共资源交易中心为中小微企业融资超过10亿元。积极促进企业创新发展，累计认定市级小企业创业基地29个、中小企业公共服务平台22个，向18家企业发放上市挂牌补贴资金1500万元。全年培育“小升规”企业200户，“个转企”完成1789户，大商集团、万达集团进入2018中国民营企业20强。

　　（五）着力加快新一轮对外开放

　　自贸区建设水平进一步提升。大连片区共复制前两批自贸区经验173项，评估形成40个制度创新案例，“保税混矿”监管创新在全国自贸区推广。全省复制推广的25个改革创新经验中大连贡献12项。大连片区进出口整体通关时间分别压缩33.3%和52.8%。

重点区域开发开放取得突破。牵头召开辽宁沿海六市联席会议，签署《辽宁沿海经济带六城市协同发展行动计划（2018-2020年）》，确定10个方面33项重点任务。实施园区创新发展等七大工程，金普新区、长兴岛经济区成为我市振兴发展的重要支撑。积极推动港口资源整合，与招商局集团正式签署了港口整合系列协议，辽宁港口集团总部落户我市，东北亚国际航运中心建设开启新里程。太平湾开发建设难题得到有效破解，将按照“前港-中区-后城”模式，着力打造东北亚“新蛇口”。

　　共建“一带一路”步伐加快。积极推进国际产能合作，在20多个国家设立服务中心、展示中心、研发中心，筹建和在建的海外仓30多个。大连港集团参与投资的吉布提国际自由贸易区正式开园，花园口经济区中英（大连）先进制造产业园建设取得新突破。积极创建“一带一路”综合试验区和中国—中东欧“16+1”经贸合作示范区。

　　招商引资和对外贸易平稳增长。制定出台45条扩大对外开放积极利用外资政策措施。印发《关于促进连台经济文化交流合作实施办法》61条，促进两地经济文化交流。加大对日韩、美欧及长三角、珠三角、京津冀地区招商力度，实际利用外资27亿美元、占全省的60%；签约国内项目协议92个，引进省外实际到位资金1057亿元、增长15.1%。高度关注、积极应对中美贸易摩擦对我市外贸的不利影响，在巩固日韩等传统市场基础上，积极拓展新兴市场，对巴西、俄罗斯出口分别增长54.4%和25.2%。精心组织参与中国首届进博会。积极推进贸易便利化，国际贸易“单一窗口”功能拓展至口岸通关全流程，综合覆盖率排名全国第二位。

沪连对口合作进一步深化。召开上海•大连对口合作第二次联席会议，确定6个方面30项合作目标任务。举办“2018沪连经贸合作上海•大连周”和第二届沪连合作论坛，开展行业对接和区域洽谈活动74场，签署经贸合作协议64个。2017年签约的57个合作项目已落实33个，落实率达57.9%。

　　（六）着力实施乡村振兴战略

　　农业发展质量显著提升。深化农业结构调整，调减籽粒玉米11.9万亩，新发展大樱桃1.7万亩，全市大樱桃种植面积达到29.8万亩、年产值超过80亿元；新建海洋牧场10万亩、国家级海洋牧场示范区9处，累计达到61.3万亩。大力促进农村一二三产业融合发展，完成5个农业产业化联合体建设，普兰店鑫玉龙示范园成为全国首家有机农业示范园。全省首个农业政策性金融创新示范市项目在庄河启动。大力开展品牌创建行动，农产品质量监测合格率达到98.9%，继续位居全国前列。全市农林牧渔及服务业增加值增长3.5%。

　　农村各项改革深入推进。加大对乡镇的政策支持力度，提升乡镇基本保障能力。实施发展壮大村级集体经济三年行动计划，53个集体经济“空壳村”经济收入实现零突破。推进农村集体产权制度改革，加大农业共营制推进力度，新组建土地股份合作社30家，土地流转面积累计达到181.5万亩，占家庭承包耕地的41.5%。全面开展“大棚房”专项清理整治行动，查处治理三大类80个违法违规项目。

　　农村基础设施日趋完善。实施农村人居环境整治三年行动，建成生活污水集中收集处理系统11套，行政村生活垃圾处理体系覆盖率达到85%，改建完成无害化卫生户厕2万座。扎实推进“四好农村路”建设，建成通屯油路411公里，大中修农村公路550公里，全市低收入村自然屯全部通上油路。开工建设3项农村饮水安全工程，解决6346户农村居民饮用水问题。

　　县域经济加快发展。出台推进县域经济发展三年行动计划及产业特色小镇创建规划，重点培育创建产业特色小镇10个。积极开展产业兴村强县示范行动，长海县荣获全国休闲农业和乡村旅游示范县称号，庄河富谷渔业成为首批创建国家农村产业融合发展示范园。瓦房店在全国“百强县”排名中继续位居东北第一。加快推进县乡财政管理体制改革，全市所有47个乡镇均保留国库。

　　（七）着力提升城市功能品质

　　基础设施建设持续推进。河口湾大桥、梭鱼湾道桥工程竣工通车，大连湾综合交通枢纽客运中心建成试运营，地铁5号线、大连湾海底隧道等一批重大基础设施项目加紧推进。马栏河运动公园、植物园改造项目建成开放。维修城市道路100条，我市成为东北地区唯一“国家公交都市建设示范城市”。加强城市排水管网改造和内涝点整治，城市防汛抗旱应急能力明显提升，庄河全国海绵城市试点建设加快推进。

　　精细化管理更加规范。构建网格化管理模式，将中心城区208平方公里区域细化为1345个日常管理网格，沙河口区获得中国大数据应用新典范服务优化奖。建立交通拥堵综合治理联席会议机制，实施城市道路交通文明畅通提升行动。持续开展市容环境综合整治，集中对卫生死角、违规占道、广告牌匾、私搭乱建、渣土运输、乱堆乱放等各类市容违法乱象进行清理。加快公共机构强制垃圾分类和示范小区建设，垃圾分类覆盖率达到30%。

　　城市文化品质不断提升。公共文化服务体系更加完善，群众性文化生活不断丰富，举办“文化有约”惠民活动500余场，“五进”公益演出172场。演出市场日益繁荣，涌现出音乐舞蹈史诗《追梦》等一批精品力作。全民阅读活动走向深入，西岗区在全国率先建成人脸识别技术“城市书房”，我市获评“中国十大数字阅读城市”。文化产业加快发展，华录集团再进“文化企业30强”。召开足球发展座谈会，制定《关于加快大连足球发展的实施意见》，我市成为全国足球改革试点城市。徒步大会、马拉松赛等体育品牌影响力不断扩大，全民健身运动深入开展。

　　（八）着力保障和增进民生福祉

　　就业实现持续增长。坚持就业优先战略和积极就业政策，全市城镇新增就业11.6万人，就业困难人员实现就业2.9万人。举办创业就业博览会等专项对接活动253场，提供就业岗位35.7万个。实现培育创业带头人1637人，带动就业10207人，组织开展职业技能培训3.2万人。

　　社保体系更加完善。全民参保计划深入实施，企业退休人员基本养老金实现十四连增，采取增收、清理不合理支出等措施，确保退休人员养老金按时足额发放。城乡居民基础养老金领取标准、低保标准、特困人员救助供养标准全面提高。为全市27187名贫困人员代缴养老保险费345.9万元。提高城镇居民基本医疗保险参保财政补助标准，支付贫困城镇居民参保人员大病保险基金638.4万元。加强退役军人服务管理工作，全力维护退役军人合法权益。发放残疾人两项补贴8083.8万元，惠及全市7.74万困难和重度残疾人。积极落实失业保险降低费率、援企稳岗等政策，为用人单位节省用工成本13.57亿元。

　　教育事业全面发展。深化教育体制改革，不断提高教育有效供给能力。全市新增公办幼儿园15所、学位3360个，新认定普惠性民办幼儿园30所。综合施策解决教育热点难点问题，全市义务教育阶段超大班额全部消除，大班额消除50%以上。新增省、市级示范性高中3所。我市获批全国青少年校园足球改革试验区，金普新区获评全国首批德育协同创新实验区。

　　卫生与健康服务水平不断提升。推进分级诊疗制度和医联体建设，共组建9个城市医疗集团、10个县域医共体和10个专科联盟，社会办医床位占比达到31.5%。加大新农合保障力度，政府补助标准提高到每人每年580元，人均基本公共卫生补助标准提高到55元。17种肿瘤靶向药品纳入医保支付范围，异地联网结算定点医疗机构达到111家。推进国家居家和社区养老服务改革试点，高标准建设中山区春海、甘井子区万科等50个居家和社区养老服务示范中心。甘井子区医疗卫生健康中心竣工，将有效缓解西部城区40万居民的就医难题。

　　社会治理稳步推进。全面落实安全生产责任，深入开展危化品等重点领域专项整治，安全生产“四项机制”经验在全国推广。全市安全生产事故起数和死亡人数分别下降23.5％和29％，2018年是我市有安全生产记录以来，首次没有较大及以上事故的年份。森林防火取得16年来最好成绩。加大食品药品监管力度，创建国家食品安全示范城市取得阶段性成果。严格落实四级书记抓信访要求，推动信访矛盾减存控增，全市信访形势总体平稳。强力开展扫黑除恶专项斗争，全市侦办黑恶势力犯罪案件102起。加强价格调控监管，严肃处理检车线违法涨价案件。依法加强宗教事务管理，集中力量梳理并解决了一批宗教领域突出问题。开展基层党建、网格化服务管理与社区警务融合联动实践，“平安大连”基础更加牢固。中山区自治、法治、德治三治融合社区模式在全国推广。

　　民族、外事、侨务、人防、档案、气象、仲裁、防灾救灾、双拥共建、民兵预备役和边海防等工作均取得新成绩。

　　（九）着力加强全面从严治党和政府自身建设

　　政治建设进一步加强。深入推进“两学一做”学习教育常态化制度化，树牢“四个意识”、增强“四个自信”、坚定“两个维护”，坚决肃清薄熙来、王珉恶劣影响，严格落实意识形态工作责任。深入开展“解放思想推动高质量发展大讨论大实践”活动，在城市未来规划、加强自贸区建设等方面形成一批研究成果、推出一批创新举措。

　　依法行政稳步实施。坚持依法科学民主决策，市直部门和区市县政府实现重大行政决策目录全覆盖。充分重视审判机关司法建议，严格公正文明执法，开展“十查十看”专项整治，行政检查压缩率59%。扎实推进行政决策、执行、管理、服务和结果公开，自觉接受人大法律监督和工作监督、政协民主监督。认真听取各民主党派、工商联、无党派人士和人民团体意见，高度重视市人大代表议案建议和政协提案办理、办结率100%。

　　工作作风持续改进。大力开展“重强抓”专项行动，严字当头，精细管理，强化实干导向、弘扬实干精神、践行实干作风，压实责任、压茬推进，挂图作战、绩效对帐。省政府180项考核任务，我市承接142项，全年目标任务完成率位居全省首位。深入开展“纠四风”“办事难”专项整治行动，推出政务服务三公开、双休日便民服务、窗口领导带班等一批便民利企措施。

　　反腐倡廉力度加大。召开政府系统党风廉政工作会议，坚持把党风廉政建设和反腐败工作摆在重要位置。坚定不移落实中央八项规定精神，认真执行《准则》《条例》，着力抓好中央、省委巡视反馈意见整改落实，建立整改长效机制。强化督查督办、考核问责和正向激励，确保党中央、国务院和省委、省政府及市委决策部署不折不扣落实到位。

　　各位代表，2018年取得的成绩来之不易!这是习近平新时代中国特色社会主义思想生动实践的结果，是党中央、国务院和省委、省政府及市委坚强领导、大力支持的结果，是改革开放40年来大连几代人不懈追求、接续奋斗的结果，是全市广大干部群众撸起袖子干出来的、挥洒汗水拼出来的。在此，向给予政府工作大力支持的人大代表和政协委员，向各民主党派、工商联和无党派人士，向离退休老同志，向中省直各部门和兄弟省市驻连机构，向驻连部队和武警官兵,向关心支持大连振兴发展的海内外朋友们，向每一位城市建设的参与者、美好生活的创造者，致以崇高的敬意和衷心的感谢！

　　总结过去一年的工作，我们清醒地认识到存在的问题和不足：一是体制机制亟待完善。“放管服”改革还不够彻底，“只进一扇门”“最多跑一次”还没有完全实现，行政审批服务信息化、网络化建设相对滞后；科技成果转化大项目不多，一些重点领域人才缺口仍然较大。二是经济结构调整任务艰巨。战略性新兴产业发展不充分、体量偏小，传统产业、传统产品占比仍然偏大；服务业转型升级需进一步加大力度；区域发展还不平衡，县域经济实力不强。三是对外开放合作需加大力度。重大开放平台建设运用不够充分，发挥对外开放拉动作用不明显；出口结构有待优化，高新技术、一般贸易、民营企业出口份额低于全国平均水平；引进外资项目储备不足。四是民生领域仍存在短板。交通拥堵、教育资源不均衡、优质医疗资源不足等问题亟待解决。五是思想观念需要进一步解放。有的干部思想观念、精神状态与新时代振兴发展要求还不适应，等靠要思想、传统路径依赖惯性思维时有表现，“不想为”“不敢为”“不会为”问题一定程度存在。我们务必高度重视，从严从实抓紧解决。

　　二、2019年政府工作目标与主要任务

　　2019年是新中国成立70周年，是全面建成小康社会的关键之年，做好各项工作意义重大。当前，我市发展面临的机遇和挑战并存。从机遇看，习近平总书记在辽宁考察时和在深入推进东北振兴座谈会上的重要讲话，为我市全面振兴、全方位振兴指明了前进方向，提供了根本遵循；党中央、国务院加大对东北振兴的支持力度，为我市加快振兴发展提供了强大动力；我国加快全方位对外开放步伐，金普新区、自贸试验区等重大开放平台进入加速发展期；恒力炼化、英特尔二期投达产，新机场、太平湾等重大项目加快建设，将为我市振兴发展提供有力支撑；深化政府机构改革，将进一步优化政府职能，提升政府治理能力，提高政府工作效率；持之以恒改进作风，各级干部作风不断转变，干事创业、担当作为的正能量正在不断聚集，共促发展、协力振兴的氛围日渐浓厚。从挑战看，国际上单边主义、贸易保护主义明显抬头，特别是中美贸易摩擦对我市经济造成的冲击和影响不可低估；国内经济运行稳中有变、变中有忧，经济下行压力加大；我市正处于转型发展的关键期，推动质量变革、效率变革、动力变革的任务十分艰巨。综合起来看，经济发展面临的环境更加严峻，不确定性更加凸显，矛盾和问题更加突出。面对机遇和挑战，我们要紧扣重要战略机遇新内涵，既要增强忧患意识，做到未雨绸缪，对面临的困难要有充分的认识，对更加复杂严峻的局面要有充分的思想准备；更要保持战略定力，坚定发展信心，在应对困难和挑战中加快振兴发展，担当起辽宁全面振兴、全方位振兴排头兵和领头羊的重要使命。

　　今年政府工作的指导思想是：坚持以习近平新时代中国特色社会主义思想为指导，全面贯彻党的十九大及十九届二中、三中全会精神，深入落实习近平总书记在辽宁考察时和在深入推进东北振兴座谈会上重要讲话精神，按照中央经济工作会议和省委、市委全会决策部署，坚持稳中求进工作总基调，坚持新发展理念，坚持推动高质量发展，坚持以供给侧结构性改革为主线，坚持深化市场化改革、扩大高水平开放，继续打好三大攻坚战，补齐拉长“四个短板”，做好“六项重点工作”，统筹推进稳增长、促改革、调结构、惠民生、防风险各项工作，进一步稳就业、稳金融、稳外贸、稳外资、稳投资、稳预期，全力保持经济持续健康发展和社会大局稳定，加快“两先区”建设，为全面建成小康社会收官打下决定性基础，以优异成绩迎接中华人民共和国成立70周年。

　　经济社会发展的主要预期目标是：地区生产总值增长6.5%左右；规模以上工业增加值增长8%以上；固定资产投资增长6%左右；社会消费品零售总额增长7%以上；外贸进出口总额占全省份额不减，力争有所提升；一般公共预算收入、城乡居民人均可支配收入与经济增长同步；城镇登记失业率控制在3.5%左右；居民消费价格指数控制在3%左右；单位GDP能耗下降3%左右。

　　为实现上述目标，要重点抓好以下几个方面工作：

　　（一）坚持推动经济高质量发展，实现全面振兴新突破

　　按照“巩固、增强、提升、畅通”要求，深化供给侧结构性改革，扎实推进“三大变革”，积极开展高质量发展试点试验，努力探索质量更高、效益更好、结构更优、优势充分释放的振兴发展新路。

　　推动制造业高质量发展。促进先进制造业和现代服务业深度融合，推动装备制造、石化等传统优势产业智能化、绿色化、品牌化发展。以大机床重整和光洋智能制造装备产业园建设为抓手，重塑“大连机床”品牌，重振“大连机床”雄风。加快金普新区智能装备产业基地建设，打造长兴岛世界级石化产业基地，加快推进恒力150万吨/年乙烯等项目建设。大力推进军民融合创新发展。培育壮大战略性新兴产业，加快人工智能、生命健康、洁净能源等产业布局，推动新一代信息技术、高端装备、新材料、新能源汽车等产业集群化发展。深入实施高新技术企业三年倍增计划，重点培育一批“瞪羚”企业和“隐形冠军”“单项冠军”企业，力争在培育“独角兽”企业上实现突破。

　　促进服务业做大做强做优。推动生产性服务业专业化、集聚化、国际化和生活性服务业便利化、精细化、品质化发展，增强对全市经济的支撑作用。充分发挥整合后的辽宁港口集团作用，以“一岛三湾”为核心，加快东北亚核心枢纽港建设。深化区港联动、多港联动，推进国家“壹港通”智慧物流跨界服务大平台示范工程,加快大窑湾、长兴岛、旅顺新港等港区重点项目建设，完善物流园区、冷链物流中心、国际邮轮母港口岸通关、配套设施和航运服务体系。完善普惠金融服务体系，强化金融对实体经济的服务支撑，放大大商所的辐射带动功能，积极引进风投、创投机构，设立地方资产管理公司。落实消费升级计划，大力推进门头房经济、特色商业街区建设，办好“慕尼黑大棚”、啤酒节、服博会、大连购物节等活动。加快一级农产品批发市场建设，加强农产品冷链物流建设，拓展城乡商贸物流配送网络，大力发展农村电商，持续释放消费潜力。发展全域旅游，推进文旅融合，做好海洋旅游和冰雪旅游的文章。引导邮轮、游艇、海岛观光、休闲垂钓等海洋旅游新业态发展，推进中国邮轮旅游发展实验区建设；做大国际温泉滑雪节、国际温泉养生文化节等品牌，促进大连旅游“淡季”升温。

　　切实稳住有效投资。坚持把招商引资作为“一号工程”，按照内资外资一起抓、国资民资一起抓、三次产业一起抓、存量增量一起抓的要求，开展精准招商、产业链招商，引进一批大项目、好项目。积极盘活闲置资源，对前期引进的重大项目，积极落实推进措施，提高项目实施效率，尽快形成更多的投资效益。加大基础设施领域补短板力度，积极盘活财政存量资金，积极争取政府债券的发行额度，确保新机场开工建设，推进人工智能、工业互联网、物联网等新型基础设施建设，推动“通信畅通工程”取得实效，加快试点5G应用，建设储能技术试点、电动汽车充电设施等重大项目。坚决落实放宽民间投资准入有关政策，积极鼓励社会资本参与政府项目，有效破解政府想干缺资金、企业想干又干不了的项目建设瓶颈问题。推动房地产业平稳健康发展，建立完善长效机制，坚持因城施策，发展住房租赁，稳定地价房价。

　　加快外贸优化升级。积极应对中美贸易摩擦，深耕日韩等传统市场，探索建立中日韩国际合作示范区和“对日经贸合作试验区”；积极拓展“一带一路”沿线国家和美洲、非洲等新兴市场。加快推进跨境电商综合试验区建设，支持我市有实力的企业在境外建立海外仓、销售网点、生产基地、研发中心等。借助夏季达沃斯论坛、中日经济合作论坛等平台，进一步提升大连国际影响力、吸引力。

　　（二）坚持深化重点领域改革，激发全面振兴新活力

　　把深化改革作为推动全面振兴的治本之策，按照“五个有利于”的要求，既谋划战略性改革，也推动战役性改革，通过改革释放内需潜力、激发经济活力、培育增长动力。

　　大力优化营商环境。着力激发微观主体活力，切实解决企业开办、运营、退出全流程各环节存在的问题，大幅提升市场主体规模，加快构建“亲”“清”新型政商关系。加大简政放权力度，全面推行政府部门权力清单、责任清单及行政审批中介服务事项清单动态管理。继续推进“双随机、一公开”和“一次检查、全面体检”，巩固“多证合一、一照一码”改革成果，全面推开“证照分离”改革。加快推进“一网、一门、一次”改革，实现“互联网＋政务服务”全覆盖。加强法治大连、诚信大连建设，制定出台《加快推进社会信用体系建设实施方案》，建立形成以信用为核心的新型市场监管机制，打造良好法治环境。深入开展营商环境专项整治，把企业的事当成亲人的事，把群众的事当成家里的事，以真心实意和真抓实干解决企业和群众反映突出的问题，让人人都是营商环境、人人建设营商环境在大连蔚然成风。

　　坚定不移推动民营经济发展壮大。认真贯彻习近平总书记在民营企业座谈会上重要讲话精神和全省加快民营企业发展大会精神，全面落实国家和省市各项支持政策，建立领导干部包扶企业机制，完善重大项目建设“服务秘书”保障制度，实现规模以上企业全覆盖，破解民营企业资金、人才、市场、科技创新等难题。进一步清理涉及民间投资管理的行政审批事项和涉企收费，切实为企业减税降费。进一步营造公平竞争环境，为民营企业创造广阔的市场空间。更加关心支持企业家成长，大力弘扬企业家精神，依法保护企业家合法权益。

　　深化国资国企改革。落实“国资国企改革年”各项举措，分类施策、一企一策，实现国有企业改革全覆盖。着力改革制约国资国企发展的体制机制，建立现代企业制度，推行有效激励的薪酬管理、职业经理人制度。全面加强国资国企党的建设，形成有效的监督管理制度体系。

　　进一步加大防范风险力度。把防控金融风险摆在更加突出的位置，严格防控政府债务风险，坚决遏制隐性债务增量，稳妥处置存量。严格防控养老金支付风险，确保养老金按时足额发放。密切关注高负债企业，做好企业信用债违约、网络借贷等风险点整治，有效防范和化解互联网金融、交易场所等风险，继续推进法治化、市场化债转股，大力化解国有企业债务。加强对中小银行监管，加大不良贷款处置力度，防止发生系统性区域性金融风险。

　　扎实推进行政管理体制改革。统筹抓好政府机构改革各项工作，聚焦发展所需，构建系统完备、科学规范、运行高效、符合大连实际的机构体系，使职能更加优化、权责更加协同、履职更加到位。稳步推进区市县机构改革，加大机构职能整合力度，构建简约高效的基层管理体制。做好事业单位改革后续工作，深化综合行政执法改革。加快全市园区整合提升，探索园区管理与开发相分离体制，在符合条件的园区开展“管委会+公司化”运营模式试点，推动形成布局合理、错位发展、功能协调的园区发展格局。

　　（三）坚持创新驱动发展战略，培育全面振兴新动能

　　主动顺应全球新一轮科技革命和产业变革大势，切实把创新摆在振兴发展的核心位置，全力推进“三创”中心建设，让全社会创新活力竞相迸发、创新源泉充分涌流。

　　加大自创区建设力度。持续推进自创区建设实施意见和三年行动计划落地落实，推动高新区、金普新区等重要承载区错位发展、特色发展。按照“又高又新”要求，全力推进自创区营造更优创新环境、集聚更多创新资源，打造具有核心竞争力的现代产业体系。探索“一区多园”发展模式，推动自创区与自贸区“双自联动”，推进自创区在科技管理体制、人才激励体制、科技金融结合等方面进行大胆探索、示范引领，实现科技创新和制度创新“双轮驱动”。

　　加快推进重大创新平台建设。积极争取洁净能源国家实验室、大连光源二期等重大科技创新平台和科学基础设施在我市布局。加快建设大连化物所洁净能源创新研究院和中科院大学能源学院、东软集团生命健康学院，继续推进大连人工智能研究院、干细胞与精准医学研究院、辽宁精细化工和催化剂产业共性技术创新平台建设。进一步提高大众创业、万众创新水平，加快国家和省“双创”示范基地建设，建立一批产业技术研发平台、产业技术创新联盟和专利联盟，办好国家双创活动周、创响中国等系列活动，激发全民创新精神和创造活力，把创新成果变成实实在在的产业活动。

　　进一步提升科技创新能力。支持大连理工大学、大连化物所建设世界一流大学和一流科研院所。在人工智能、先进装备制造、洁净能源、集成电路等13个先导型重点产业领域进行精准布局，组织实施科技重大专项、重点科技研发计划和科技创新基金。落实好支持科技企业创新的优惠政策，发挥财政投入引导和撬动作用。开展科技创新项目化管理，提升科技成果本地转化率。推进国家技术转移东部中心大连分中心、大连技术专利公开交易市场建设。实行最严格的知识产权保护，加强知识产权运用，形成有效的创新激励机制。全市高新技术企业达1400家，技术合同交易额达190亿元。

　　完善人才发展机制。深入实施高层次人才创新、科技人才创业、重点领域创新团队支持计划和城市发展紧缺人才、海外优秀专家引进计划，打造领军人才培养、博士后人才集聚和高技能人才开发等工程，完善政府特殊津贴、外国人才来华工作许可证制度，提升人力资源服务市场化和专业化水平，突出对重大科技贡献、优秀创新团队和青年人才的奖励激励，让各类人才在大连安心、安身、安业。大力培育“工匠精神”，每年评定一批“大连工匠”，打造知识型、技能型、创新型劳动者队伍。

　　（四）坚持统筹区域协调发展，构建全面振兴新格局

　　立足我市资源禀赋、功能定位，实施陆海统筹、城乡联动，扎实推进“西拓北进”战略，加快形成全域统筹、竞争有序、绿色协调、共享共赢的区域协调发展新机制。

　　全面提升城市功能品质。突出规划引领，科学编制《大连2049城市愿景规划》，启动至2035年的城市空间规划编制，描绘新时代大连高质量发展的美好蓝图。推进重大基础设施建设，加快大连湾海底隧道和光明路延伸工程、金普城际铁路、大连北站综合交通枢纽、地铁4号和5号线等项目建设；启动梭鱼湾公园改造工程，实施道路照明升级改造。做足城市更新“绣花”功夫，继续推进垃圾强制分类管理，扩大居民垃圾分类试点实施范围，完善末端处置设施建设；推进厕所革命，城镇新建改造公厕125座、新增内厕开放169座；推进五好小区建设、停车场建设和城市绿化，全面提升城市精细化、智慧化管理水平，打造更加干净、有序、美丽、宜居的城市环境。

　　深入实施乡村振兴战略。认真贯彻落实中央农村工作会议精神，坚持农业农村优先发展，全面落实“五个振兴”总要求。深化农业供给侧结构性改革，推动一二三产业融合发展，发展农产品精深加工，推进复州城农业产业强镇示范建设；加强农产品地理标志培育，大力推广“大连大樱桃”“大连苹果”等形象标识。开展青年农场主培训，培养一批爱农业、懂技术、善经营的职业农民。加快美丽乡村建设，加大农村环境综合整治力度，持续推进农村生活垃圾分类减量，改建无害化卫生户厕16.3万座。抓好“四好农村路”建设，新建通屯油路350公里，大中修农村公路400公里。推进新一轮农村改革，深化农村土地制度、集体产权制度改革，积极发展农民合作经济组织，支持村级集体经济发展壮大，消灭集体经济“空壳村”100个以上。以敢啃硬骨头的精神，继续开展“大棚房”专项清理整治，确保3月底前完成任务。

　　持续壮大县域经济实力。贯彻落实全省县域经济发展三年行动计划，推动产业向北转移、基础设施向北延伸、生产要素向北集聚。以重点园区为载体，培育引进一批重大产业项目及配套企业，培育壮大特色产业，增强县域经济综合实力。坚持产城融合，开展“飞地经济”试点，推动“飞地园区”建设。加大财税支持县域经济力度，增强县乡两级财政实力，扩大县域经济发展自主权。加强中心镇和特色小镇建设，提升小城镇公共服务水平和承载能力，促进创业集聚、就业集聚、居住集聚。

　　加快打造海洋经济强市。研究制定加强国家海洋中心城市建设指导意见，大力发展海洋渔业、海洋交通运输业、海洋旅游业、海洋船舶业、海洋工程装备制造业等现代海洋经济五大主导产业，加快发展海洋生物制药、海洋清洁能源、海洋新材料等新兴产业，积极推进海洋信息服务、涉海金融保险、涉海法律服务等海洋服务业。高质量建设现代海洋牧场，叫响“大连海鲜”品牌，打造“蓝色粮仓”，推动我市由海洋资源大市向海洋经济强市转变。

　　（五）坚持加强生态环境建设，打造全面振兴新优势

　　牢固树立绿水青山就是金山银山、冰天雪地也是金山银山的理念，以更高标准打造美丽宜居典范城市，努力让大连天更蓝、山更绿、水更清、生态环境更美好。

　　大力加强生态环境保护。构建以生态保护红线、环境质量底线、资源利用上线和环境准入负面清单为主要内容的“三线一单”环保管控模式，强化环境保护硬约束。加强海域海岛岸线保护，加大岸线治理修复力度，完善生态补偿机制。开展林业生态建设工程，全年完成营造林14万亩。修订《大连市环境保护条例》和制定《大连市海洋环境保护条例》等地方性法规，为加强生态环境保护提供法治保障。

　　坚决打赢污染防治攻坚战。巩固蓝天保卫战成果，提升扬尘精细化管控水平，启动露天矿山扬尘综合整治，加大秸秆资源化利用力度，全年空气优良天数保持在300天以上。强力推进碧水工程，深入实施河（库）长制，推进饮用水水源地规范化建设，加强海上污染源控制、入海排污口日常监管、重点海域污染整治。加强公共治污设施建设，推进农村乡镇生活污水处理厂和管网建设。全面实施净土工程，实行污染地块风险分级，开展国土绿化行动，有序修复被破坏的山体、河流、湿地、植被和废旧矿山，增加城市生态总量。

深入抓好环保督察整改。建立健全环保督察反馈问题整改常态化机制，坚持问题导向、目标导向、成果导向，对中央环保督察“回头看”和国家海洋督察反馈问题以及群众信访投诉问题不回避、不遮掩，严格对标对表，一个节点一个节点地狠抓落实，对正在推进的整改项目，以最高标准、最严要求，确保按时保质完成。

　　全面推进绿色发展。大力构建绿色产业体系，提高节能、环保、资源循环利用等绿色产业技术装备水平，积极推广装配式建筑。加快推进能源结构调整和能源资源全面节约，构建清洁、安全、高效的能源体系，稳步推进清洁取暖。

　　（六）坚持发挥开放龙头作用，建设全面振兴新高地

　　实施更加积极主动的开放战略，以高质量开放推进高质量发展，以全面开放引领全面振兴，让高水平开放成为大连最鲜明的城市品格。

　　高标准建设自由贸易试验区。加快落实辽宁自贸区总体方案确定的119项任务和大连片区实施方案确定的234项任务，着力在投资贸易便利化、金融开放创新、事中事后监管等方面争取更大的先行先试权，探索更多创新经验，走在全国前列。全力申建自由贸易港，对标国际投资和贸易规则体系，围绕监管、投资、贸易、航运、金融、税收等制度创新，推进准自由港先行先试，构筑开放层次更高、营商环境更优、辐射作用更强的开放新高地，在东北振兴发展中发挥引擎作用、体现大连担当、做出大连贡献。

　　打造辽宁沿海经济带升级版。充分发挥核心和龙头作用，进一步完善沿海经济带六市合作机制，着力推进基础设施互联互通、港口资源整合、市场要素对接、产业协同协作、公共服务共建共享等深度合作。强化产业支撑，培育壮大石化、高端装备等临港产业集群。全力推进港口整合，加强港口间分工合作、错位发展，协同打造现代化辽宁港口集群，推动太平湾“港产城创”一体化建设迈上新台阶。

　　深度融入共建“一带一路”。积极创建“一带一路”综合试验区和中国－中东欧“16+1”经贸合作示范区。加强与沿线国家港口、铁路、内陆港站交流合作，推进“辽满欧”“辽海欧”等国际物流大通道建设，支持中欧班列扩大运营规模。加强国际产能合作，完善综合服务体系和风险防范机制，助力装备、技术、标准、服务“走出去”。加快中英（大连）先进制造产业园建设。

积极对接国家区域发展战略。持续深化沪连对口合作，推进科技创新、人才交流等领域务实合作，加强装备制造、港航物流等产业对口合作，探索结对合作、园区共建等创新合作模式。全面做好东西部扶贫协作和对口支援，建立长效机制，强化精准帮扶、结对帮扶，加快实施产业合作项目，拓展帮扶领域和内容。

　　（七）坚持在发展中保障和改善民生，共享全面振兴新成果

　　坚持尽力而为、量力而行，扎实推进15项重点民生工程，用心用情用力解决人民群众所需所急所盼，让人民群众的获得感更充实、幸福感更真实、安全感更踏实。

　　把稳就业摆在突出位置。完善“六个一批”工作机制，着力解决好高校毕业生、农民工、退役军人等重点群体就业，全年实现城镇新增就业8万人。大力实施失业保险降费率、失业保险费返还、失业保险参保职工提升技能补贴等政策，积极预防和有效调控失业风险。持续开展贫困家庭子女等重点群体的就业技能培训、创业培训和岗位技能培训，确保零就业家庭动态清零。鼓励以创业带动就业，力争让每个想创业、会创业、能创业的劳动者找到人生出彩的舞台。

　　健全完善社会保障体系。织密扎牢社会保障网，加快建立统一的城乡居民医疗保障制度。继续实施提高城乡居民收入三年行动计划，持续提高各类群体收入水平。根据国家相关政策，适时提高企业、机关事业单位退休人员基本养老金标准。全面实施全民参保计划,推进社会保险法定人员全覆盖。完善社会救助体系，持续提高城乡低保、特困人员救助供养标准。让振兴发展成果更多更公平惠及全市人民。

　　提升社会事业发展水平。深化教育教学改革，着力提升各类教育质量。新增公办幼儿园15所、公办普惠学位6000个。推进义务教育优质均衡发展，新建义务教育学校10所，适应新高考改革需要，促进普通高中多样化特色发展。深化产教融合、校企合作，完善职业教育体系。推动师德建设常态化、长效化，完善中小学教师待遇保障机制。全面建设健康大连，推进医养结合和健康城市国家试点。深化医药卫生体制改革，落实分级诊疗制度，推进医联体建设，提升基层医疗卫生服务能力。推进国家级居家和社区养老服务改革试点，继续推动居家和社区养老服务示范中心建设。鼓励社会力量兴办教育、医疗、养老等社会事业。持续推进燃气管网改造工程，新增天然气用户9.8万户。深入实施安居工程，持续开展老旧住宅、老旧电梯维修改造，支持引导老旧住宅加装电梯，改善市民居住品质。继续抓好“暖房子”“菜篮子”、城市供暖、过街地下通道管理等重点民生工程，持续解决好办证难、回迁难问题，干一件成一件，一抓到底，善作善成。

　　增强城市文化软实力。践行社会主义核心价值观，弘扬新时代辽宁精神。完善公共文化服务体系，丰富群众文化生活，繁荣文艺精品创作，开通“文化大连云”平台，高水准规划建设中国传媒岛。加强文物保护利用和文化遗产保护传承，加快推进东关街、太阳沟等区域规划编制和保护利用。积极发展壮大文化旅游、文化创意产业。举办高端体育赛事，深入开展全民健身活动。重振“足球城”雄风，加快建设体育强市，增强城市凝聚力，汇集振兴正能量。推进文明城市创建常态化、长效化，努力实现全国文明城市“六连冠”，让文明创建成果造福人民群众。

　　坚决打好精准脱贫攻坚战。加大政策、项目、资金倾斜力度，推动产业、教育、健康、就业、社保兜底等扶贫举措有效落地，聚焦重点，精准施策，挂图作战，积极稳妥推进低收入村脱贫退出“清零行动”，确保全市低收入村摘帽51个，让越来越多的群众尽快摆脱贫困、过上幸福生活。

　　构建更高水准的平安大连。深入开展扫黑除恶专项斗争，依法打击和惩治各类违法犯罪活动。加快推进“云上公安•智慧警务”建设，全面完成“雪亮工程”。认真落实“七五”普法规划，加强人民调解员队伍建设。深入开展安全生产大检查和专项整治活动，推进安全发展示范城市创建工作，强化风险预警和应急管理体系建设。加强食品药品监管，严厉打击制售假冒伪劣食品药品等违法犯罪行为。坚持四级书记抓信访，扎实推进信访矛盾减存控增三年攻坚行动。提高社区治理和服务水平，打造共建共治共享社会治理新格局。

认真开展第四次全国经济普查和第九轮全国双拥模范城创建工作。继续做好民族、宗教、外事、侨务、对台、人防、档案、气象、仲裁、防灾减灾、民兵预备役及退役军人等各项工作。

　　（八）坚持严格落实管党治党责任，展现全面振兴新作为

　　切实把政治建设摆在首位，加强全面从严治党，进一步树牢“四个意识”、增强“四个自信”、坚定“两个维护”，确保中央大政方针和省委、省政府及市委决策部署落地见效。

　　深入推进思想解放。扎实开展“解放思想推动高质量发展大讨论大实践”活动，坚持“仰望天空”与“脚踏实地”相结合，深入查找“四个短板”的具体表现，在高质量发展实践中找答案、寻方法、解难题。坚决做到“七破七立”，上海等先进城市敢想、敢干的，我们也要敢想、敢干，不断消除僵化陈旧的思维定势、摆脱传统路径依赖惯性，切实以思想上的破冰带来行动上的突围、实践上的创新。

　　加快建设法治政府。坚持依法行政，不断增强法治理念和法治意识，带头尊法守法用法，充分发挥法律顾问与公职律师的作用，把政府工作全面纳入法治轨道，稳步推进行政执法“三项制度”。依法接受人大法律监督、工作监督，主动接受政协民主监督，推进政治协商，认真办理人大代表议案建议和政协提案，自觉接受司法监督、舆论监督和社会监督。推进审计监督全覆盖。

　　持之以恒改进作风。深入开展“重强抓”专项行动，坚持“严细实”的工作作风，推进各项工作落到实处、取得实效。政府要带头过紧日子，让人民过上好日子，大力压减一般性支出，严控“三公”经费预算，除重点和刚性支出外，其他领域一般性支出压缩10%。大力倡导“契约精神”，企业有要求、群众有诉求，政府必须有回应；凡是部署的任务、承诺的事情，政府必须说到做到、守信践诺，令行禁止、雷厉风行，切实增强政府公信力和执行力，建设人民满意的服务型政府。

　　坚持反腐败无禁区全覆盖零容忍。落实党风廉政建设和反腐败工作三年计划，深入推进政府系统党风廉政建设和反腐败工作。严格执行中央八项规定精神及实施细则，严肃查处顶风违纪行为，坚决防止“四风”反弹回潮。强化权力运行监督制约，围绕权力运行关键环节，健全廉政建设与业务工作融合机制，严格落实依法科学民主决策程序，加大对公共资金、国资国企、公共资源交易等重点领域监管。健全容错机制，强化正向激励，努力营造海晏河清的政治生态，激发干事创业的昂扬激情，形成振兴发展的强大合力。

　　各位代表，新的一年，市政府将牢记习近平总书记的嘱托，不负全市人民的重托，从我做起、率先垂范，坚决做到信念坚定、为民服务、勤政务实、敢于担当、清正廉洁，并带动全市各级政府恪尽职守、励精图治、奋勇争先，努力创造无愧于党、无愧于人民、无愧于时代的新业绩。

　　各位代表，幸福都是奋斗出来的！我们每个人为这座城市多献一份爱、多出一份力、多流一把汗，就能形成百川汇海、聚沙成塔的强大合力，我们的家乡一定会变得更加美好，大连全面振兴的宏伟蓝图一定能够如期实现。让我们更加紧密地团结在以习近平同志为核心的党中央周围，高举习近平新时代中国特色社会主义思想伟大旗帜，在市委的坚强领导下，满怀激情、团结奉献，锐意进取、埋头苦干，以新气象新担当新作为，加快推进“两先区”建设，奋力谱写大连全面振兴、全方位振兴的壮美新篇章。
